# Supplementary material for: Previously hidden landslide processes revealed using distributed acoustic sensing with nanostrain-rate sensitivity
Source: Nat Commun. 2024 Jul 24;15:6239. doi: 10.1038/s41467-024-50604-6 (PMC11266418; doi:10.1038/s41467-024-50604-6)
Supplement: Supplementary file 1 — Supplementary Information [file 41467_2024_50604_MOESM1_ESM.pdf]

Supplementary Materials for

Previously hidden landslide processes revealed using distributed acoustic sensing with nanostrain-rate sensitivity

Susanne Ouellet *et al.*

*Corresponding author. Email: susanne.ouellet2@ucalgary.ca

This PDF file includes:

Supplementary Figs. S1 to S8

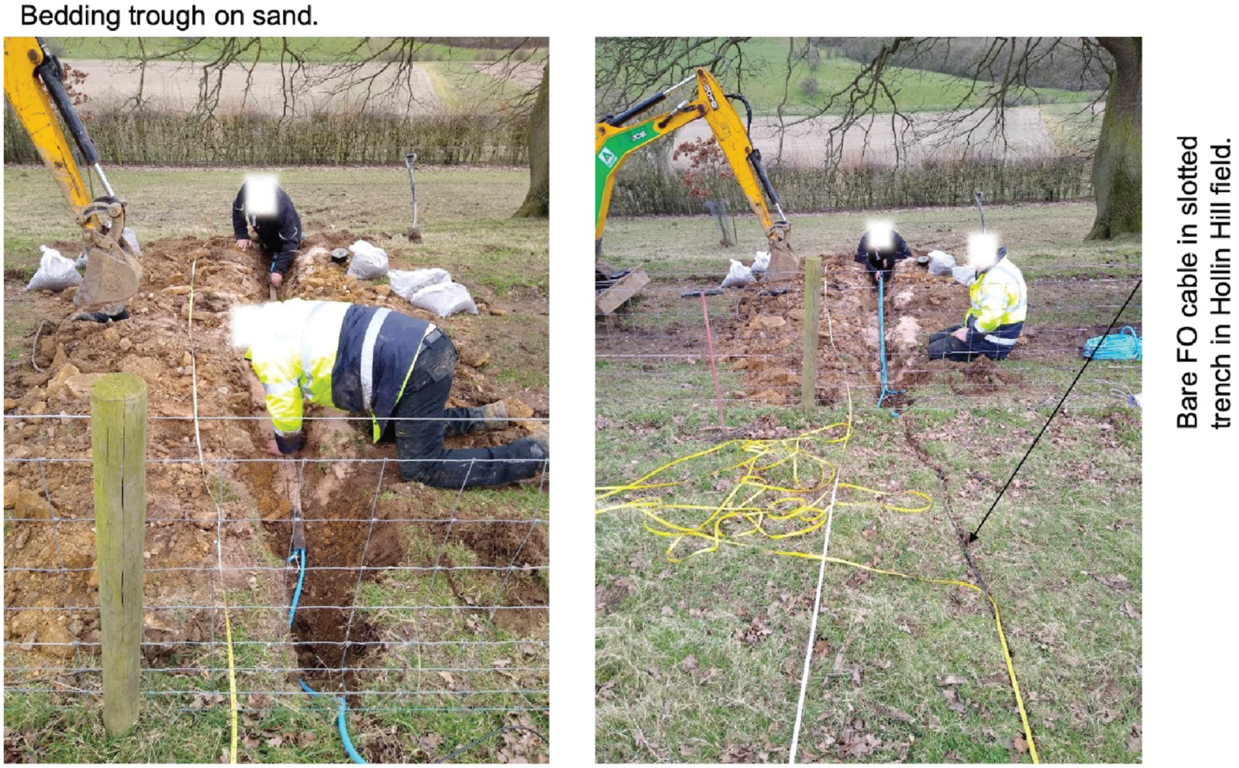

Fig. S1 Photos of the fiber optic installation, showing the transition from protective housing to the bare cable at Hollin Hill. Faces have been blurred to conceal identities of individuals in photograph.

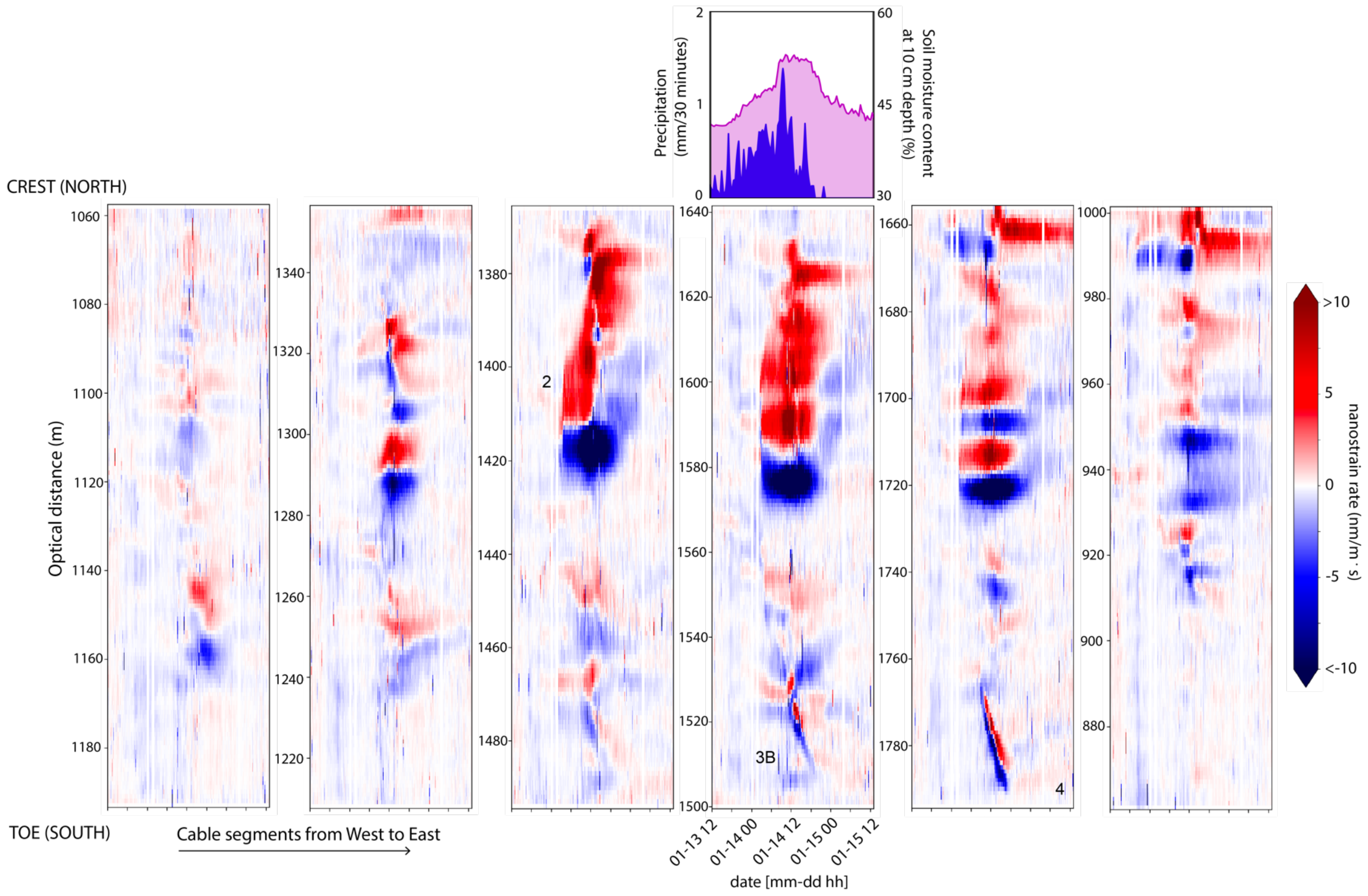

Fig. S2 DAS strain-rate spatiotemporal images along each cable section (labelled L1 to L6, westward to eastward). Channels represent the optical distance (in ~meters) along the fiber. Precipitation and soil moisture data shown above cable section L4 for comparison.

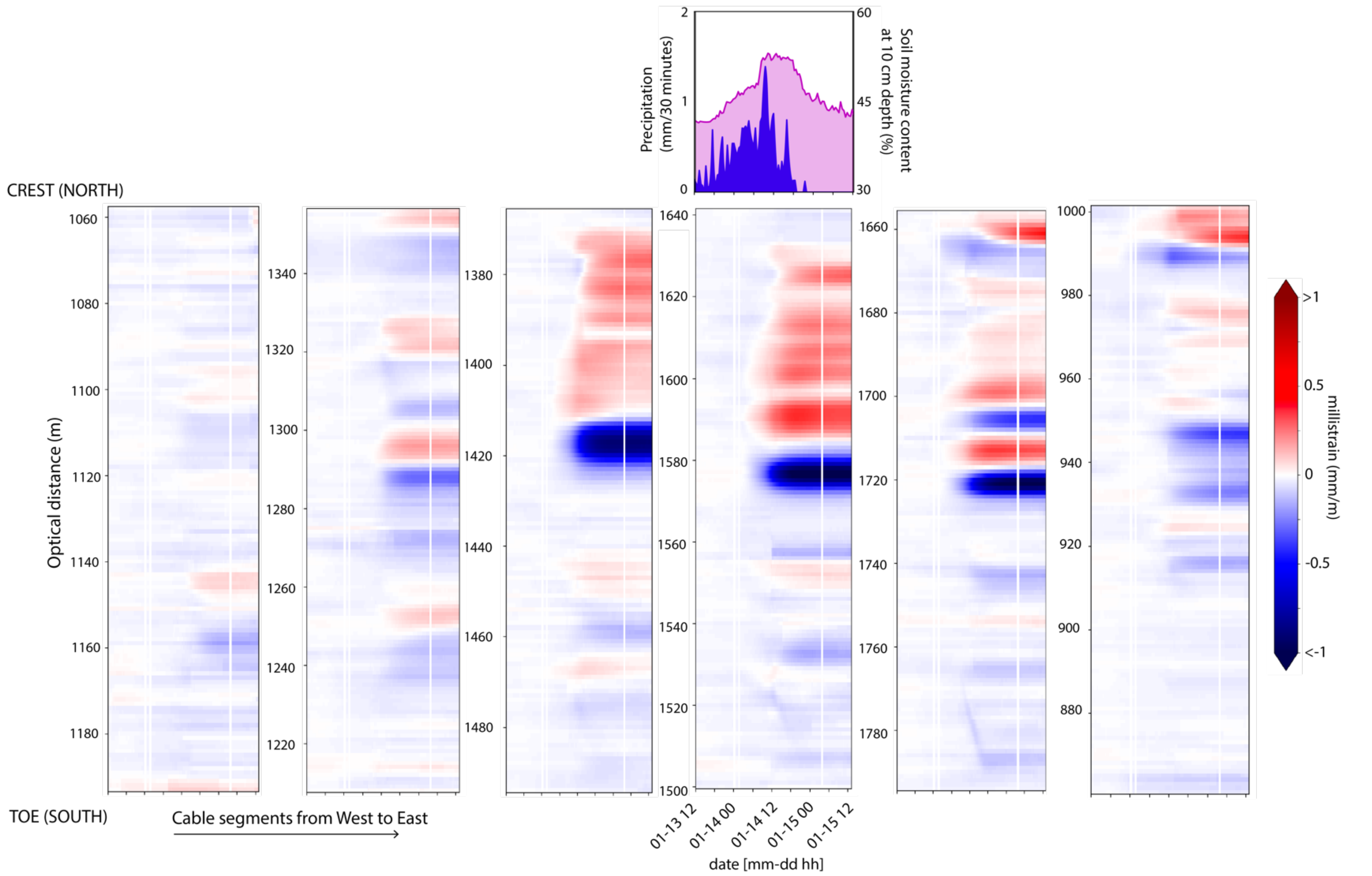

Fig. S3 DAS strain spatiotemporal images along each cable section (labelled L1 to L6, westward to eastward). Channels represent the optical distance (in ~meters) along the fiber. Precipitation and soil moisture data shown above cable section L4 for comparison.

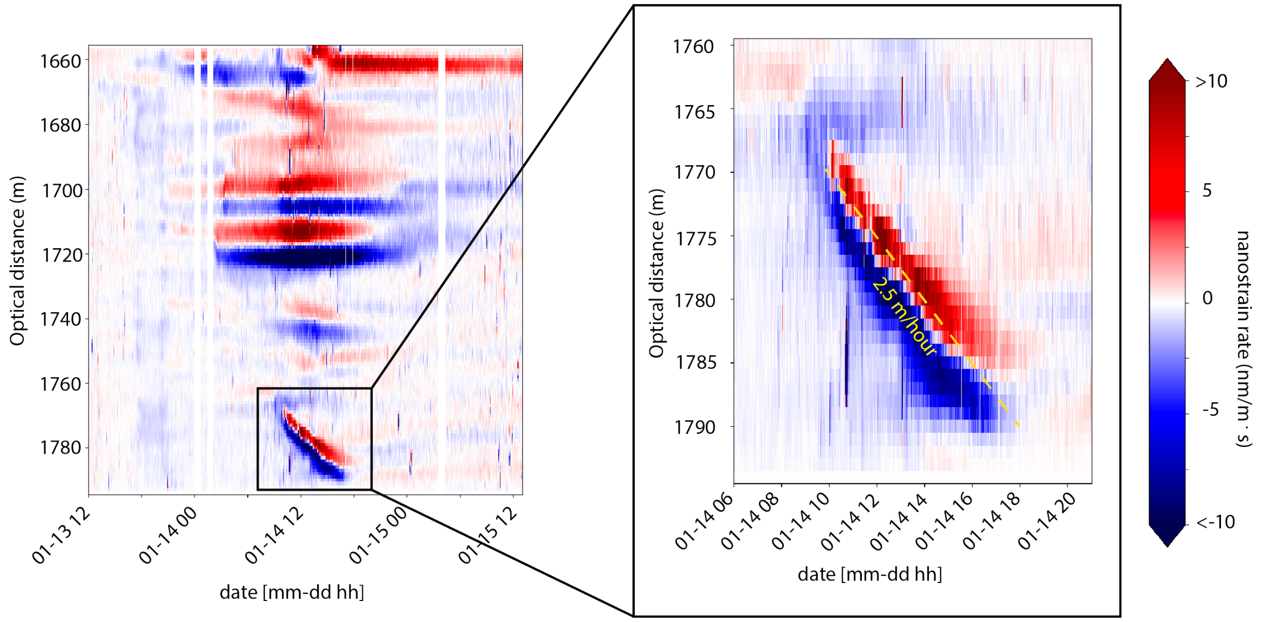

Fig. S4. DAS strain rate spatiotemporal image at cable section L5. Inset figure highlights the speed of propagation of the strain rate front, at ~2.5 m/ hour.

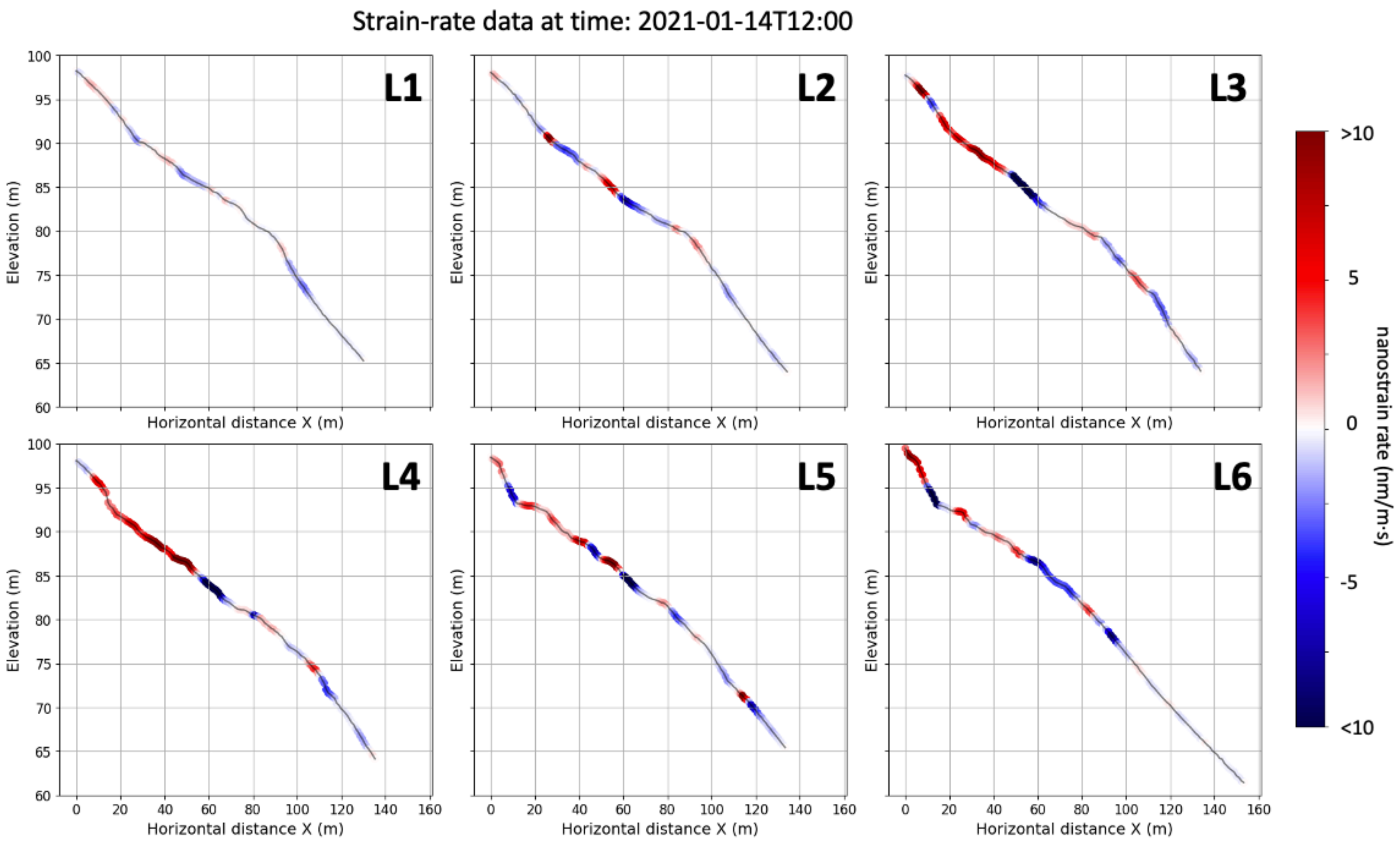

Fig. S5. DAS strain-rate data overlain onto topographic sections based on ground lidar survey acquired in November 2020 (labelled L1 to L6, westward to eastward).

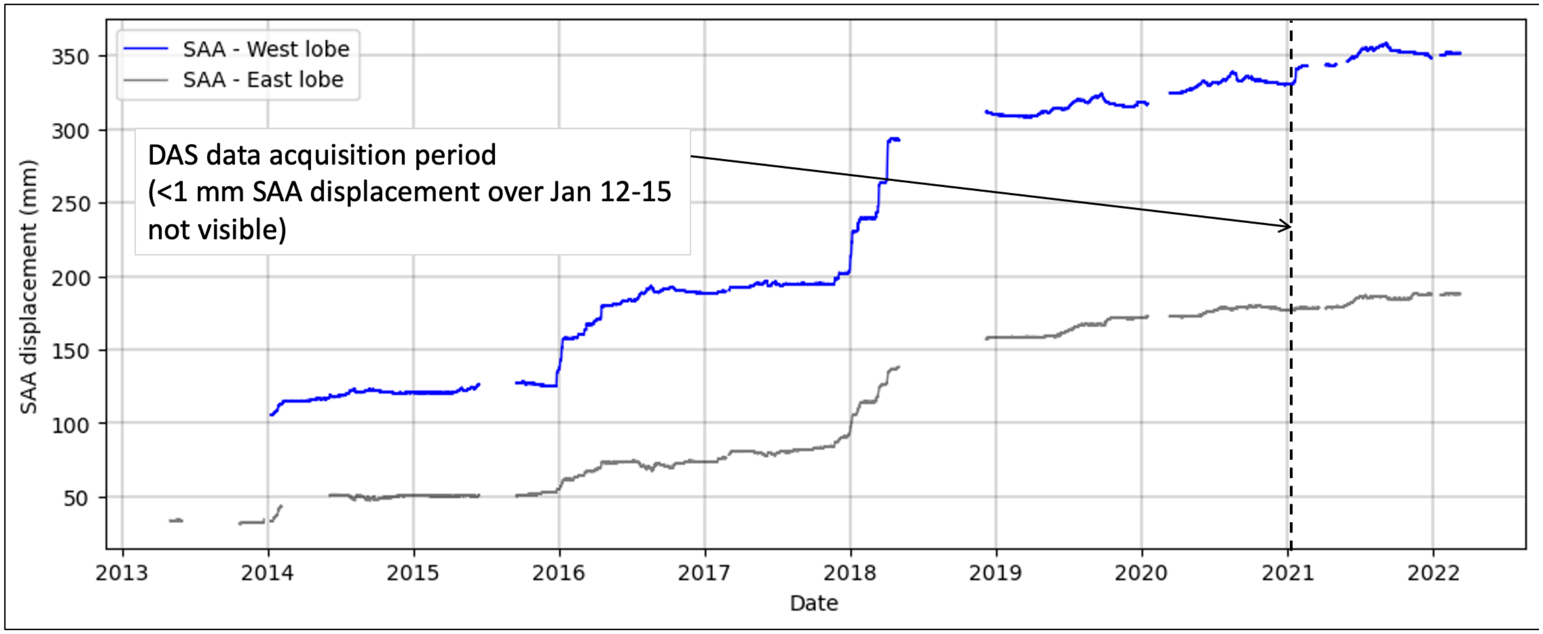

Fig. S6 West and east lope ShapeArray displacement from installation in 2013 to 2022. DAS data acquisition period (January 12-15 2021) shown as dashed line to illustrate insignificant displacement (<1 mm) considering overall displacement trends.

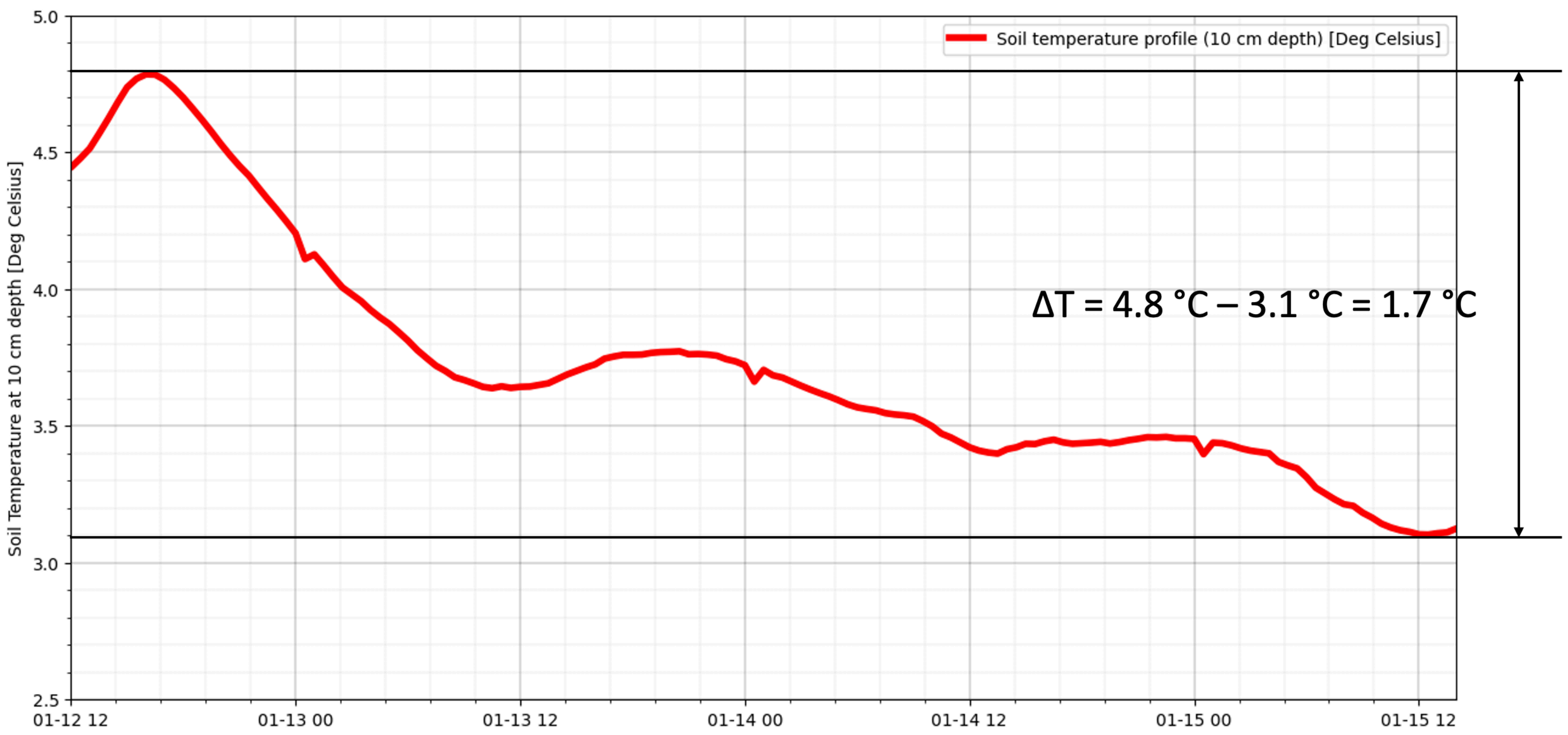

Fig. S7 Soil temperature sensor installed at 10 cm depth over the three-day DAS acquisition period.

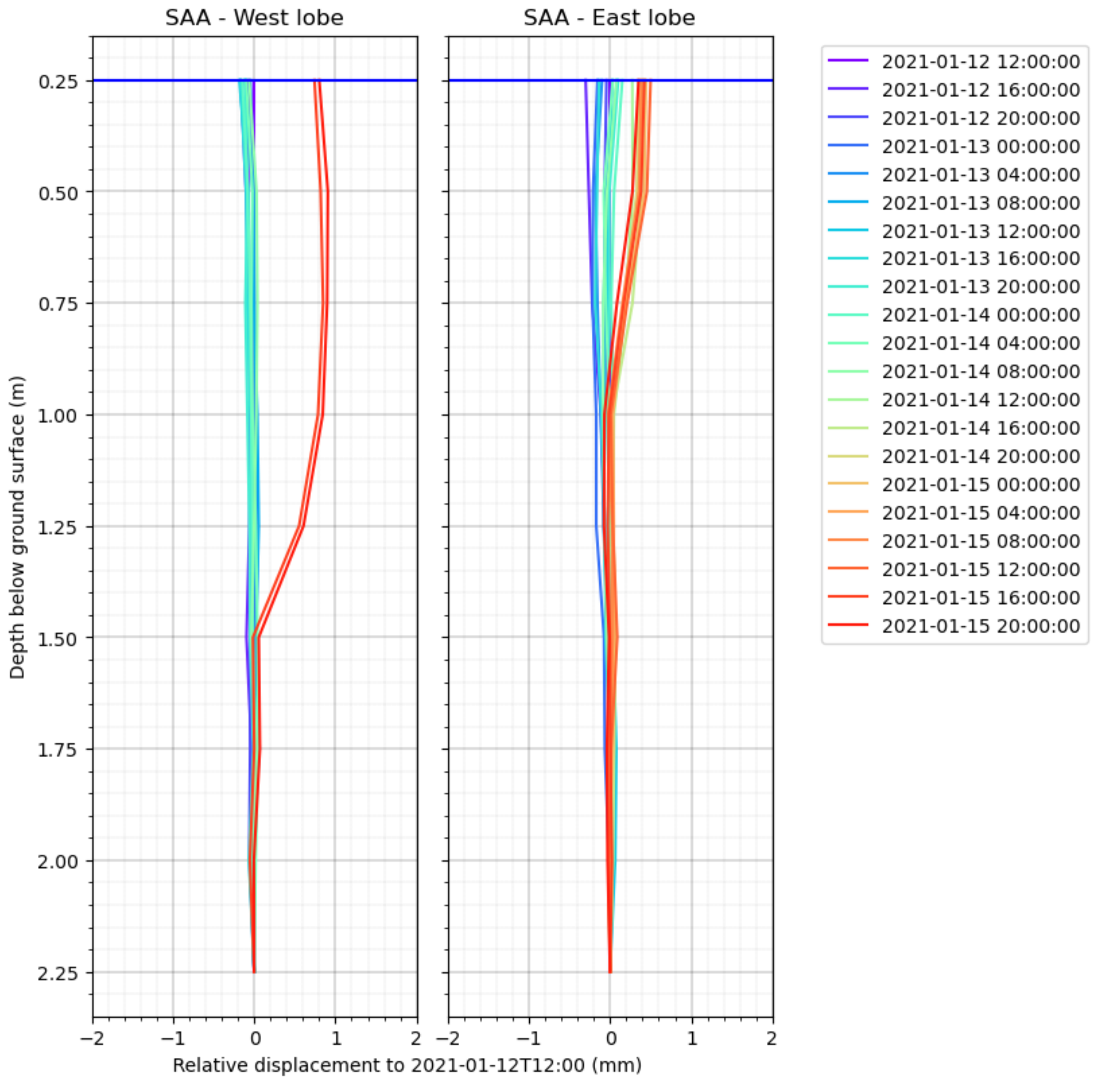

Fig. S8 SAA displacement profiles for west and east lobe showing change in displacement over DAS data acquisition period.
